# Integrated geotechnical and mineralogical evaluation of the subgrade of some failed pavements along Enugu-Onitsha expressway Southeastern Nigeria

**DOI:** 10.1038/s41598-023-41289-w

**Published:** 2023-08-26

**Authors:** Kelechi Paulinus Ukor, Ogbonnaya Igwe, Obialo Solomon Onwuka, Stella Kosi Nzereogu, Kelechi Nnaji Eze, Pearl Elochukwu Echezona

**Affiliations:** Department of Geology, University of Nigeria, Nsukka, Enugu Nigeria

**Keywords:** Environmental sciences, Geology

## Abstract

This research is focused on the constant pavement failure in parts of the Enugu-Onitsha expressway. The failed portions are constructed on the natural subgrade known to be the Shale of Enugu and Mamu formations southeastern Nigeria. Five samples each were obtained from the formations and were subjected to geotechnical test, mineralogical analysis, scanning electron microscopy (SEM) and, statistical analysis. The outcome of the geotechnical test revealed that the liquid limits (LL) are of high plasticity with values ranging from 57.69 to 62.61% and 53.57 to 56.24%, plasticity index (PI) values ranging from 20.32 to 24.37% and 13.37 to 15.32%, Slake durability index (SDI) values ranging from 0.55 to 31.8% and 63.4 to 71.6%, for Enugu and Mamu Shales respectively. From the Unconfined compressive strength (UCS) test, the cohesion (C) values ranged from 37.36 to 43.89 kPa and 24.22 to 27.07 kPa, soaked California bearing ratio (CBR) values ranged from 1.03 to 1.22% and 0.90 to 1.60% for Enugu and Mamu Shales, respectively. The test results are not in the range of specification of the Nigerian Federal Ministry of Works and Housing (FMWH) Nigeria standard for pavement construction. X-ray diffraction (XRD) analysis revealed major minerals to be quartz and kaolinite. Moreover, kaolinite disperses and migrates when moist hence geotechnical failure. Images from Scanning electron microscopy revealed the presence of micropores and fractures which can be used as an inference to the geotechnical test results. Statistical analysis of key geotechnical parameters such as SDI, PI, C, and CBR revealed a strong correlation either positively or negatively with each other. The evaluated results pointed out that the underlined natural subgrade is poor for pavement construction, and thus requires improvement.

## Introduction

Standard means of transportation especially road networks is considered to be of topmost importance in a nation’s economy. Nigeria as a nation is not left out as its major means of transportation is via road network. In addition, development and sustainability of good road and highway networks in these parts of the world (i.e., the developing countries like Nigeria) are difficult, and has led to economic backwardness in that sector^1^. According to Aghamelu et al.^1^, damaged roads are caused by unavailability of construction materials and poor construction methods. Research by Akudo et al.^2^ suggested the fact that a good high way network is a huge positive parallelism between a country’s economic increases. It is therefore important that a country’s high way network should be planned and modeled in a professional and attractive way so as to efficiently utilize the value of the public and the economic satisfactory assistance. Several casualties including death of humans and loss of asset costing fortunes are being displaced due to vehicular accidents as a result of damaged road networks. Researches done by Owoyemi et al.^3^ outlined some important causes of failed roads, these include geological, geomorphological, lack of drainage, geotechnical, road usage, faulty designs, plan and model inadequacies, and inadequate maintenance factors. However Okogbue et al.^4^, and Aghamelu et al.^1^ had observed that pavement designs and other engineering/civil structures had failed and suffered much destruction soon after construction has been completed due to weak foundation the design is built on, and constant maintenance of such design failure is very costly.

The highway is an engineering structure composed of layers (i.e., subgrade, subbase, and base course) in flexible pavement designs. The foundation soil is also the subgrade soil and the outcome of the in-situ weathering of the basement rock. Thus it is an outcome of geology and the climatic factor of an environment. Unfortunately, geological factors are not often taken seriously during design and rehabilitation of several highways in Nigeria even though the subgrade soil is an outcome of geology^5^. Thus the model of the highway must embrace these factors, mainly climate and geology as they influence the outlook and how durable the road would be in a long term.

Naturally the Geology of a particular area really determines the durability of any road construction. Before the design of any road construction, the properties of the subgrade (in-situ soil) of that particular area is evaluated critically, because majority of the difficulties faced by contractors during road construction is majorly the potential of the subgrade which serves as the foundation for road construction, and if not suitable it cannot bear the load placed on it. If this be the case then it is very certain that after the construction, road failure is inevitable^6^. According to Maduka et al.^7^, highway pavement standard is not only dependent on the properties of the natural subgrade on site, but the geology of the area and the construction materials to be used.Despite the great dissatisfaction towards the properties of shale for construction purpose^1^, and Onyeobi et al.^8^ made a proposition that in the lower Benue trough, the tertiary shales located mostly in the eastern part of Nigeria has displayed an inadequate execution when used as foundational soil (subgrade) for pavement construction and other civil engineering designs due to their properties.

**Figure 1:**
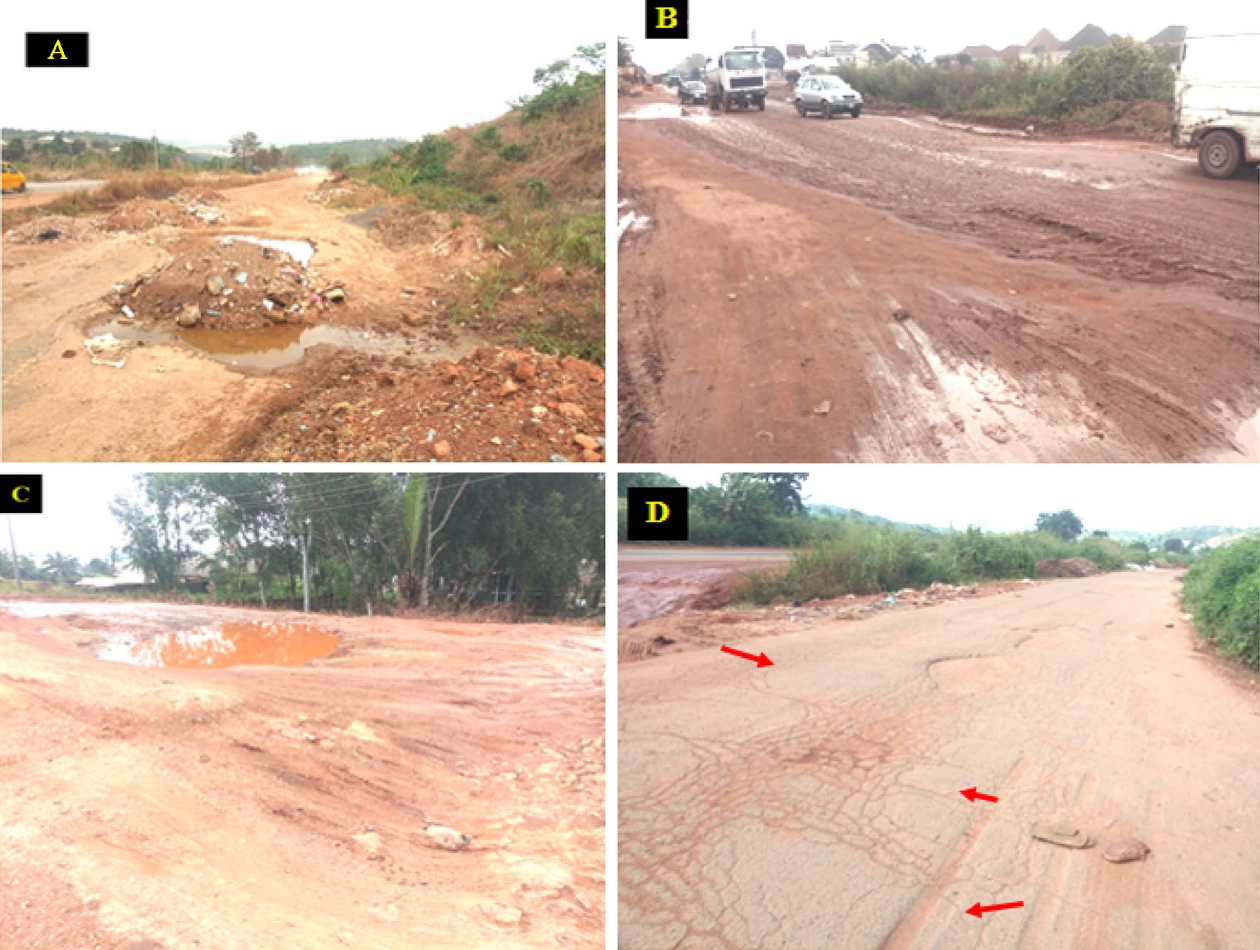
(**A**–**D**) Showing failed pavements along Km 26–45 Enugu-Onitsha Expressway Southeast Nigeria.

Enugu-Onitsha express way, which is a major south eastern road network that links the western states and some part of southern states to the eastern part of Nigeria for over six years have suffered immeasurable damage despite repeated reconstruction of failed portion (Fig. 1). This is because the likely cause (s) of the consistent failure is (are) yet to be understood and rehabilitation exercise without proper understanding of the micro pores and structures of the subgrade soil will continue to trigger financial damage and wastage of construction materials. Hence this present study combines geotechnical and geochemical investigation to evaluate the natural subgrade along some failed portion of Enugu-Onitsha Express way Southeastern Nigeria both as a rock and soil material. The specific objectives were to, determine the characteristics behavior of the natural subgrade for engineering purpose; evaluate the relationship among mineralogy, micro fractures, micropores and other selected geotechnical properties; compare the results obtained from the analysis with Nigerian Federal Ministry of Works and Housing Nigeria (FMWH) standard for subgrade material for pavement construction, and correlate the relationship among key geotechnical parameters such as SDI (slake durability index), PI (Plasticity index), CBR (California Bearing Ratio), and c (Cohesion).

## Location and physiography of the study area

Enugu-Onitsha expressway is a dual carriage expressway that runs from latitude 6° 27′ 00″ N to 6° 29′ 00″ N and Longitude 7° 27′ 00″ E to 7° 29′ 00″ E. The compass direction of the study area is trending in a north-southern direction, having a high topography to the western region with an elevation of about 400 m above sea level that continues around the Milliken hill area and Udi plateau southeastern Nigeria. The study area experiences a very heavy downpour of rainfall which originates from the tropical rain forest region’s climates, accompanied by a stubborn high-intensity storm that ranges annually between 1850 and 2500 mm. There are two prevalent seasons the study area experiences which include the wet and dry season respectively^9^. The wet season begins in April and gradually increases in intensity through May and June with a dramatic drop in August (commonly referred to as the August break). It later continues to down pour accompanied by ranging storms, resuming from late August to October^10^. The study area displays a dendritic drainage pattern, which is drained by the Iva, Ekulu, Nyaba, and Ogbete Rivers that flow towards the eastern part of the Cross River Basin. The geomorphorlogical characteristics of the drainage within the study area are that it is poorly drained in the eastern region and well drained towards the western region.

## Geology

**Figure 2:**
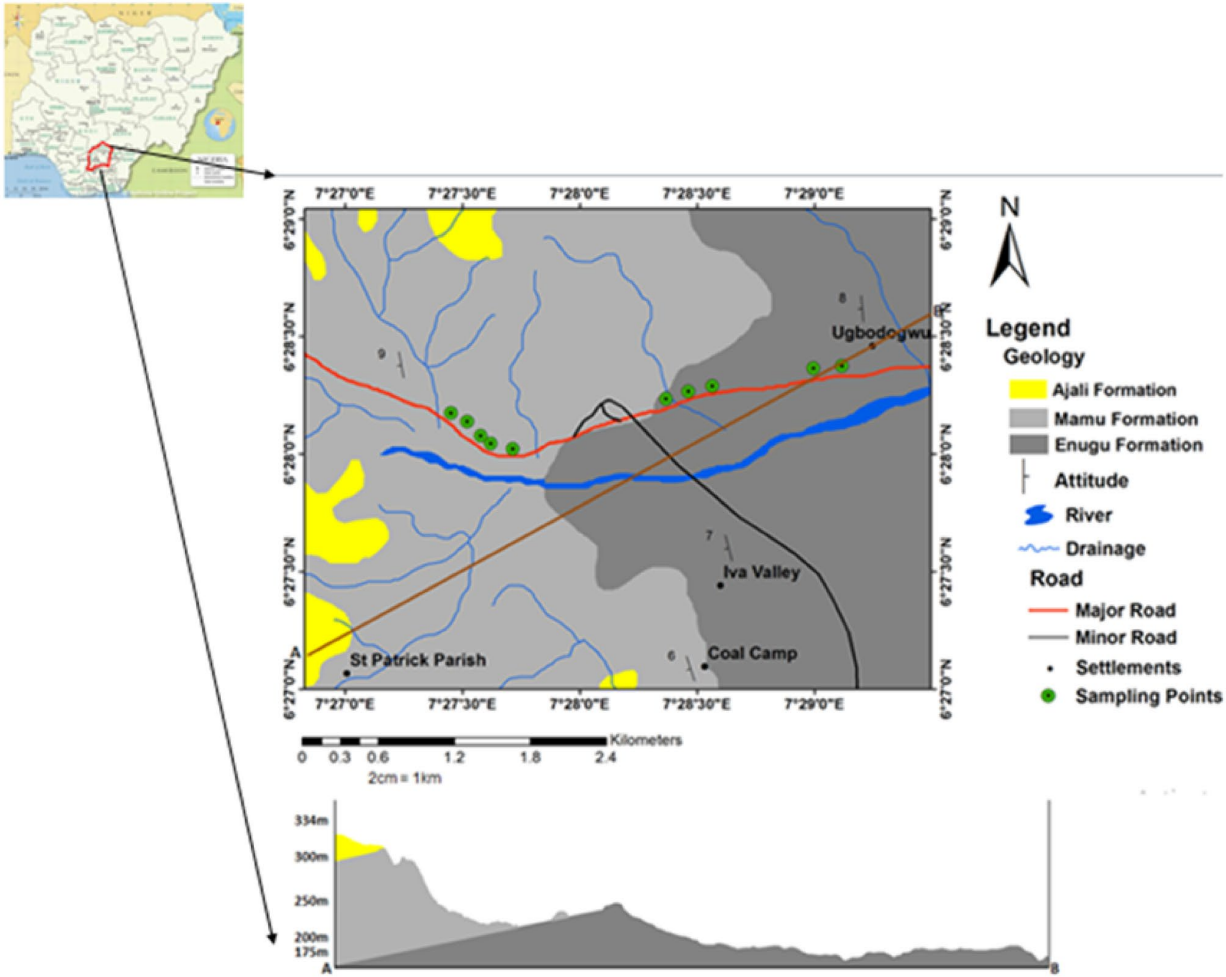
Geologic map of the study area.

Enugu-Onitsha is located within the Anambra Basin, where its mode of occurrence can be traced or derived from the late Santonian tectonic event within the Benue trough that led to the origin of a new sedimentary basin. The study area is underlain by three major geologic Formations; Enugu, Mamu and Ajali formations, but more emphasis was laid on the Enugu and Mamu formations. According to Nwajide^11^, the Enugu formation overlays directly above the Awgu formation (Fig. 2). Additionally the Enugu Formation (shale) is a lateral extension of the Nkporo/Owelli formation and also said to be among the oldest deposit in the Anambra Basin It is made up greyish fissile shale accompanied by ironstone with the presence of pyrite capped above. The Mamu formation is said to be a restricted formation in the Anambra Basin and Afikpo Syncline in southeastern Nigeria consisting of interbeds of siltstone, shale, heteroliths, and fine grained sands with coal seams Igwe^12^. Research by Reyment^13^ revealed that piles of sediment vary across the basin ranging from 75 to over 1000 m in different parts of the basin, which was said to be deposited on an estuarine floodplain, tidal flat floodplain and swamp under paralic conditions. The Maastrichtian Ajali Sandstone formation lies conformably on the Mamu Formation with its type locality along the valley of the Ajali River near Enugu, Kogbe^14^. Previous work on these has revealed it to be friable and dated to be of Campanian to Maastrichtian age, deposited in a shallow marine environment.

## Methodology

During this stage, the natural subgrades were identified, described, and the attitude of outcropping beds was measured and recorded. A total of ten freshly disturbed shale samples were obtained and utilized for this study. Five samples each from different formations (Enugu and Mamu respectively). With the help of a 6 inch hand auger, sampling spacing of 5 m, and depths ranging from 0.5 to 3.5 m, the samples were collected successfully. The ten shale samples were stored in an airtight nylon bag to retain its natural moisture before they got to the laboratory for analysis. Ten fresh samples were taken to the Civil Engineering Laboratory, University of Nigeria Nsukka and were subjected to California Bearing Ratio (CBR), Atterberg consistency limit (LL, PL and PI), and Unconfined compressive strength. The CBR tests were performed on compacted samples in soaked conditions, following the procedure of BS 1377 part 4^15^, using a hammer weighed 4.89 kg and giving 56 evenly distributed blows to compact the soil for the test procedure. However, soaking was done overnight (24 h) in a water-filled bathtub, as suggested by Okagbue and Ochulor^16^ to determine the behavior of the subgrade in worst-case scenario during the wet season. Atterberg Consistency Limit, and Unconfined Compressive Strength tests were done according to the appropriate codes (D4318 and D2166) of the American Society for Testing and Materials (ASTM)^17^.The liquid limit and plastic limit tests were carried out on air-dried samples that passed that passes the 425-µm (No. 40) sieve. The unconfined compressive strength of the samples was tested with zero confining pressure, The UCS values at 15% strain were used to determine the strength (cohesion) of the sample materials. Ten samples were taken to the University of Port Harcourt, Nigeria for the Slake durability index (SDI) test and X-ray diffraction (XRD). The SDI values were determined after the test conducted on each group was repeated for 2 cycles was done following the appropriate codes (ASTM D4644). The X-ray diffraction (XRD) was done by directing an x-ray beam to the sample, after which the scattered intensity as a function of the outgoing direction was then measured. After the beam was separated, the scatter (also referred to as the diffraction pattern), revealed the sample’s crystalline structure. Scanning electron microscopy (SEM) imaging of the shale samples was analyzed at the Obafemi Awolowo University, Nigeria. The SEM machine scans a focused electron beam over the surface of the sample to create an image. The key geotechnical parameters such as plasticity index (PI), slake durability index (SDI), California bearing ratio (CBR), and cohesion (c) were correlated with each other. Similarly, the relationship between the geotechnical properties was analyzed using Pearson’s correlation matrix.

## Results and discussion

### Field observation

**Figure 3:**
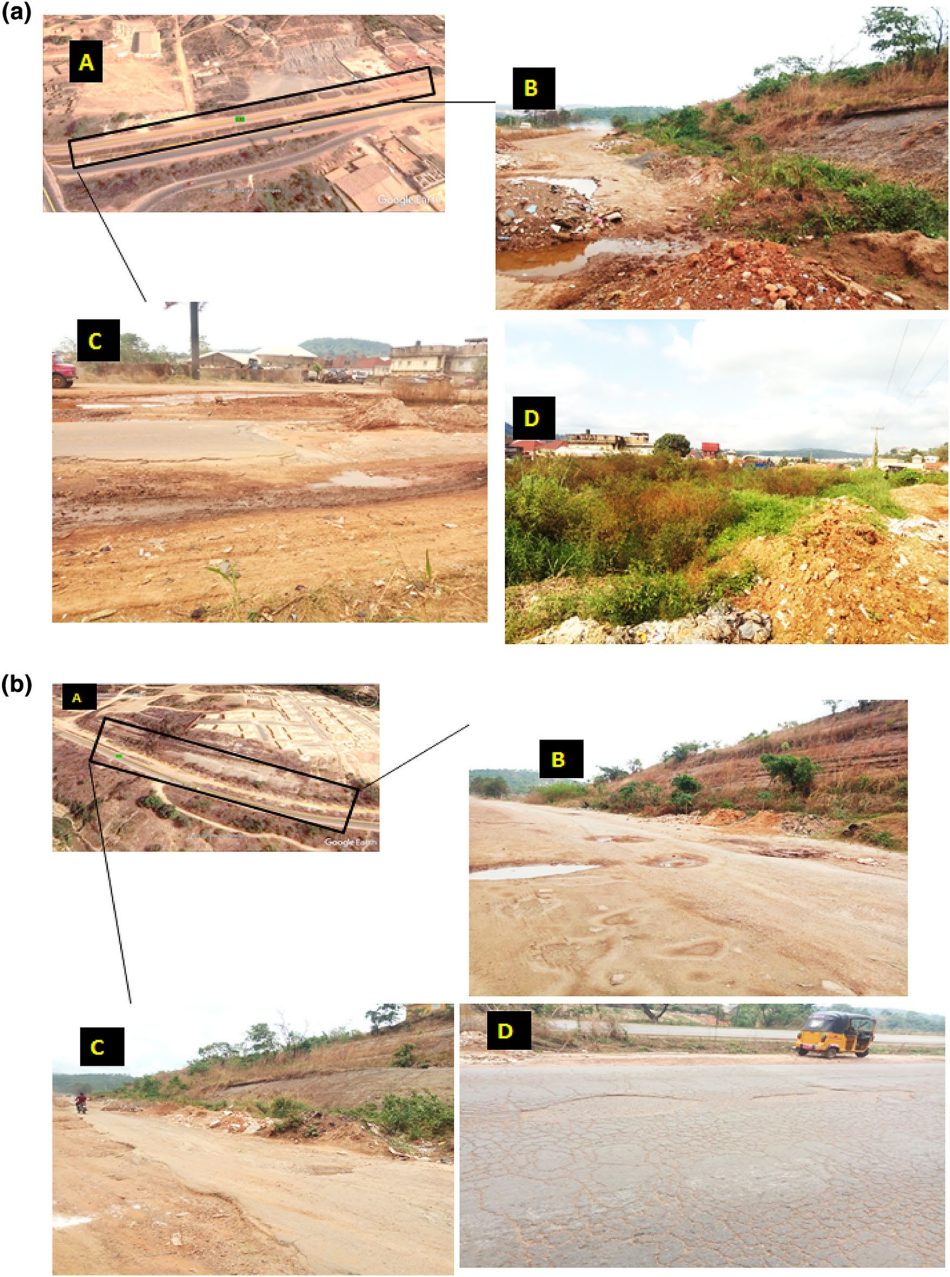
(**a)** (**A**) Google earth image of the failed pavement underlained by Enugu Formation. (**B**) Field photograph of deformed/abandoned portion of the expressway Km 36. (**C**) Field photograph of deformed pavement at Ugbodogwu (Enugu-Onitsha expressway Km 36). (**D**) field photograph of abandoned failed section of Enugu Onitsha expressway gradually turning into a dump site (Km 37). (**b**) (**A**) Google earth image of the failed pavement underlained by Mamu Formation; (**B**) field photograph of deformed/abandoned pavement with portholes; (**C**) field photograph of deformed pavement leading to Ninth mile; (**D**) field photograph of portholes and major cracks along stable pavement underlained by the Mamu Formation along Enugu-Onitsha expressway Km 41.

Detailed field work commences with basic geological descriptions, i.e., the basic observations that a geologist would make through direct field observation. Such observations gave information on the location, outcrop, and current state of the pavement. Cracks, portholes, and deformations of various kinds were observed in the study area (Fig. 3a(a–d);b(a–d)). Detailed field mapping revealed that the study area was underlain by three major formations, namely Enugu, Mamu, and Ajali formations, but more emphasis was laid on the failed pavements underlain by the Enugu and Mamu formations respectively.

### Soil indices

#### Atterberg limit

**Figure 4:**
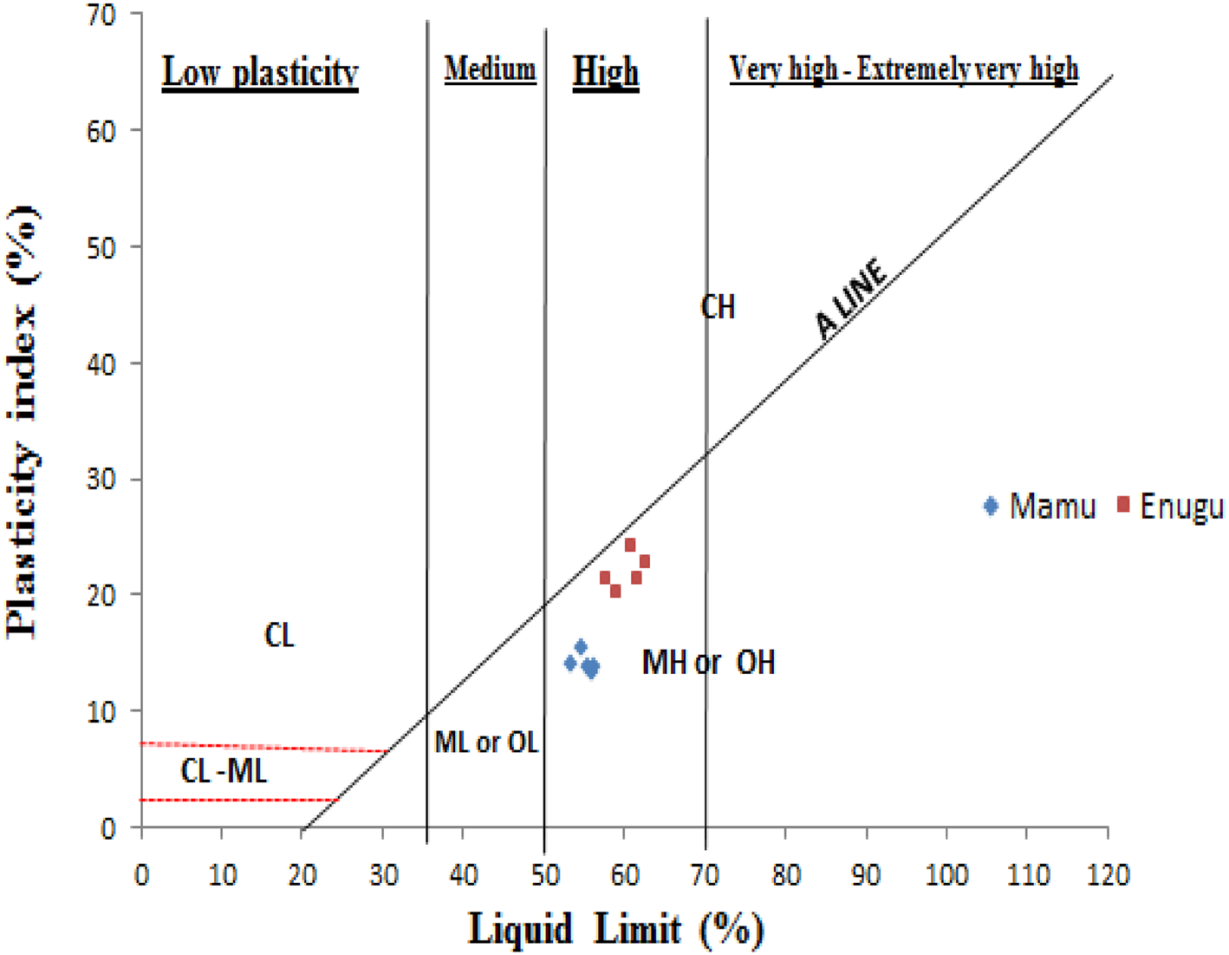
Plasticity chart (after^20^).

From the results obtained, the LL and PL of Enugu Shale values ranged from 57.6 to 62.6% and 35.64 to 40.00%, while those of Mamu Shales were in the range of 53.57 to 56.24% and 39.39 to 42.67%, respectively. Both Shale samples LL were classified as materials with high plasticity according to Bell^18^ liquid limit classification (Tables 1, 2). Enugu Shale had PI values ranging from 20.32 to 24.37%, which is significantly higher than that of Mamu Shale, with values ranging from 13.37 to 15.32%, respectively. The PI values of Enugu Shale were classified to be of medium swelling potential and that of Mamu to be of low swelling potential according to Ola^19^ classification of the potential expansiveness of soil, making use of the material PI values (Tables 3, 4). The Casagrande plasticity index chart^20^ revealed that all samples fell below the A-line as seen in (Fig. 4). According to the unified soil classification system (USCS), the samples plotted below the A-line greater than 50% are termed organic soil (OH) with high plasticity. Following the Nigerian Federal Ministry of Works and Housing Nigeria (FMWH)^21^ standard for subgrade, LL < 30% and PI < 13% respectively, both Shale samples did not conform to the set standard laid down by FMWH. In this light, both samples are poor subgrade materials and will cause pavement failure if constructed on them. An evaluation of the similarities and differences between the sample results and that of the FMWH for road and bridge design is displayed in (Table 5).

**Table 1:** Classification for liquid limit after^18^.

Description	Plasticity	Range of liquid limit
Lean or silty	Low plasticity	< 35
Intermediate	Intermediate plasticity	35–50
Fat	High plasticity	50–70
Very fat	very high plasticity	70–90
Extra fat	Extra high plasticity	> 90

**Table 2:** Atterberg limit test result for LL.

Shale ID	Liquid limit (%)	Plasticity	AASHTO classifications/subgrade rating	USCS classification
E1	60.76	High plasticity	A-7-6/fair to poor subgrade	OH
E2	62.61	High plasticity	A-7-6/fair to poor subgrade	OH
E3	61.48	High plasticity	A-7-6/fair to poor subgrade	OH
E4	57.69	High plasticity	A-7-6/fair to poor subgrade	OH
E5	58.78	High plasticity	A-7-6/fair to poor subgrade	OH
M1	54.71	High plasticity	A-7-6/fair to poor subgrade	OH
M2	53.57	High plasticity	A-7-6/fair to poor subgrade	OH
M3	55.44	High plasticity	A-7-6/fair to poor subgrade	OH
M4	56.24	High plasticity	A-7-6/fair to poor subgrade	OH
M5	56.03	High plasticity	A-7-6/fair to poor subgrade	OH

*E* Enugu Shale, *M* Mamu Shale,* OH* organic clay*.*

**Table 3:** Potential expansiveness of soils after^19^.

Plasticity index (%)	Swelling potential
0–15	Low
16–25	Medium
26–35	High
> 35	Very high

**Table 4:** Atterberg limit test result for PI.

Shale ID	Plasticity index (%)	Swelling potential
E1	25.12	Medium
E2	22.98	Medium
E3	21.48	Medium
E4	21.46	Medium
E5	20.32	Medium
M1	15.32	Low
M2	14.13	Low
M3	13.73	Low
M4	13.70	Low
M5	13.37	Low

*E* Enugu Shale,* M* Mamu Shale.

**Table 5:** Comparison of key geotechnical parameters of a subgrade with Federal Ministry of Works and Housing standard.

Parameters	LL (%)	PI (%)	SDI (%)	Soaked CBR (%)	Remarks
FMWH	30	13	80	5–11	Suitable
E1	60.76	25.12	0.55	1.03	Not suitable
E 2	62.61	22.98	1.70	1.16	Not suitable
E 3	61.48	21.48	10.30	1.22	Not suitable
E 4	57.69	21.46	18.10	1.22	Not suitable
E 5	58.78	20.32	31.80	1.33	Not suitable
M 1	54.71	15.32	69.6	0.90	Not suitable
M 2	53.57	14.13	63.4	1.00	Not suitable
M3	55.44	13.73	67.1	1.06	Not suitable
M 4	56.24	13.70	71.6	1.32	Not suitable
M 5	56.03	13.37	71.3	1.60	Not suitable

*E* Enugu Shale,* M* Mamu Shale*.*

#### Slake durability index (SDI)

The SDI was done to estimate the long-term performance and durability of the material as a rock. The outcome of the slake durability index test according to the (ASTM D4644) standard revealed that Mamu Shale had values ranging from 63.4 to 71.6%, which was significantly higher than that of Enugu Shale, with values ranging from 0.55 to 31.8% (Table 6). These were also in correspondence with the range of durability index of various rock types according to Underwood et al*.*^22^. According to the slake durability index classification rating by Franklin and Chadara^23^, Mamu Shale was rated medium while Enugu Shale was rated very low to low (Table 7). The SDI values for both shale samples are not in conformity with the recommended standard for subgrade, which is > 80% according to the Nigeria specification for road and bridge design^21^, thus they are below standard and are not suitable as a subgrade for pavement construction except if stabilized properly. An evaluation of the similarities and differences between the sample results and that of the Nigeria specification for road and bridge design is displayed in Table 5.

**Table 6:** Slake Durability Index test result.

Sample ID	Slake durability index(SDI)%	Class of durability
E1	0.55	Very low
E2	1.7	Low
E3	10.3	Very low
E4	18.1	Very low
E5	31.8	Very low
M1	69.6	Medium
M2	63.4	Medium
M3	67.1	Medium
M4	71.6	Medium
M5	71.3	Medium

*E* Enugu Shale,* M* Mamu Shale*.*

**Table 7:** Slake Durability Index classification after^23^.

Percentage	Durability classification
0–25	Very low
26–50	Low
51–75	Medium
76–90	High
91–95	Very high
96–100	Extremly high

#### Unconfined compressive strength (UCS)

**Figure 5:**
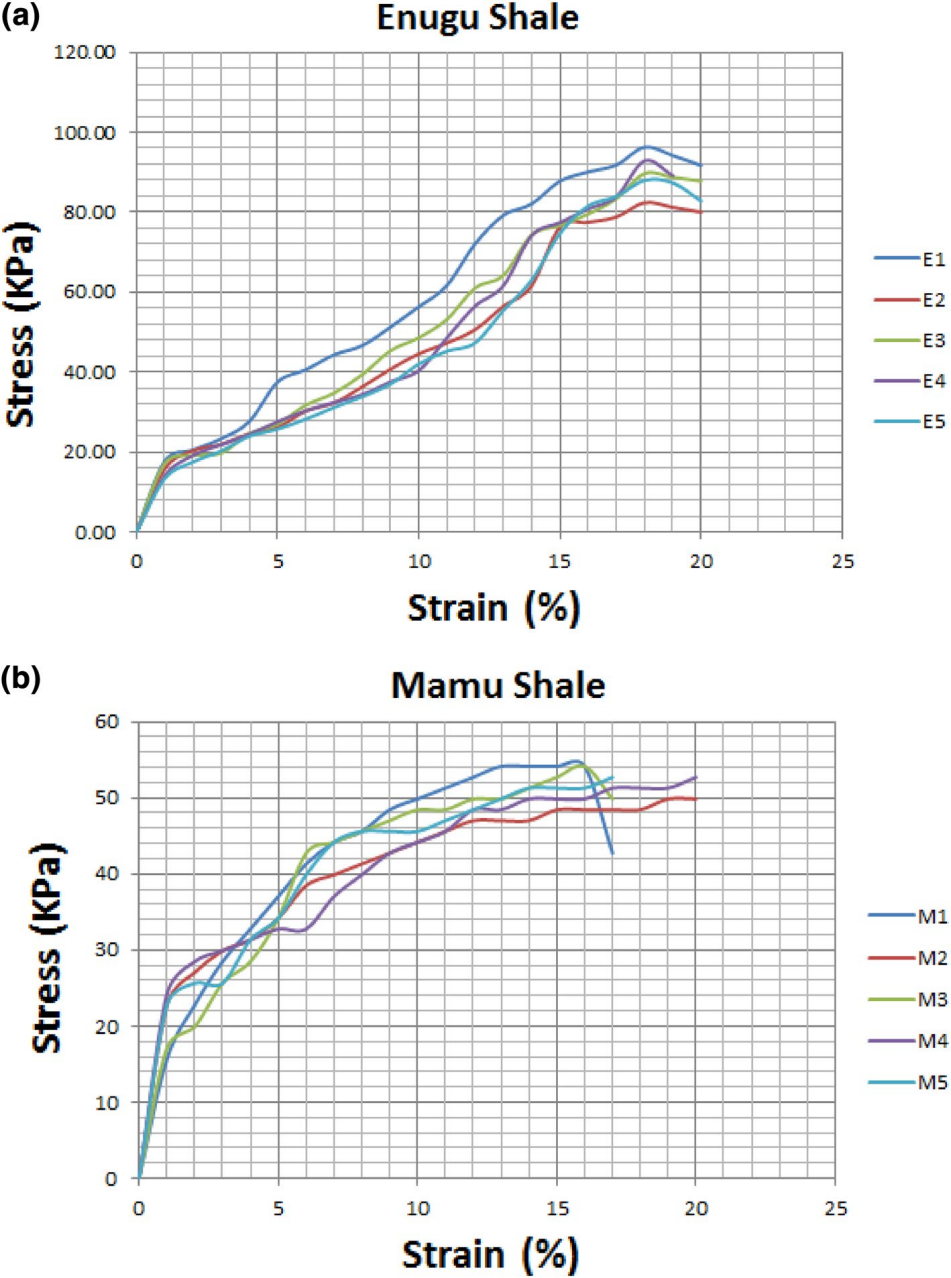

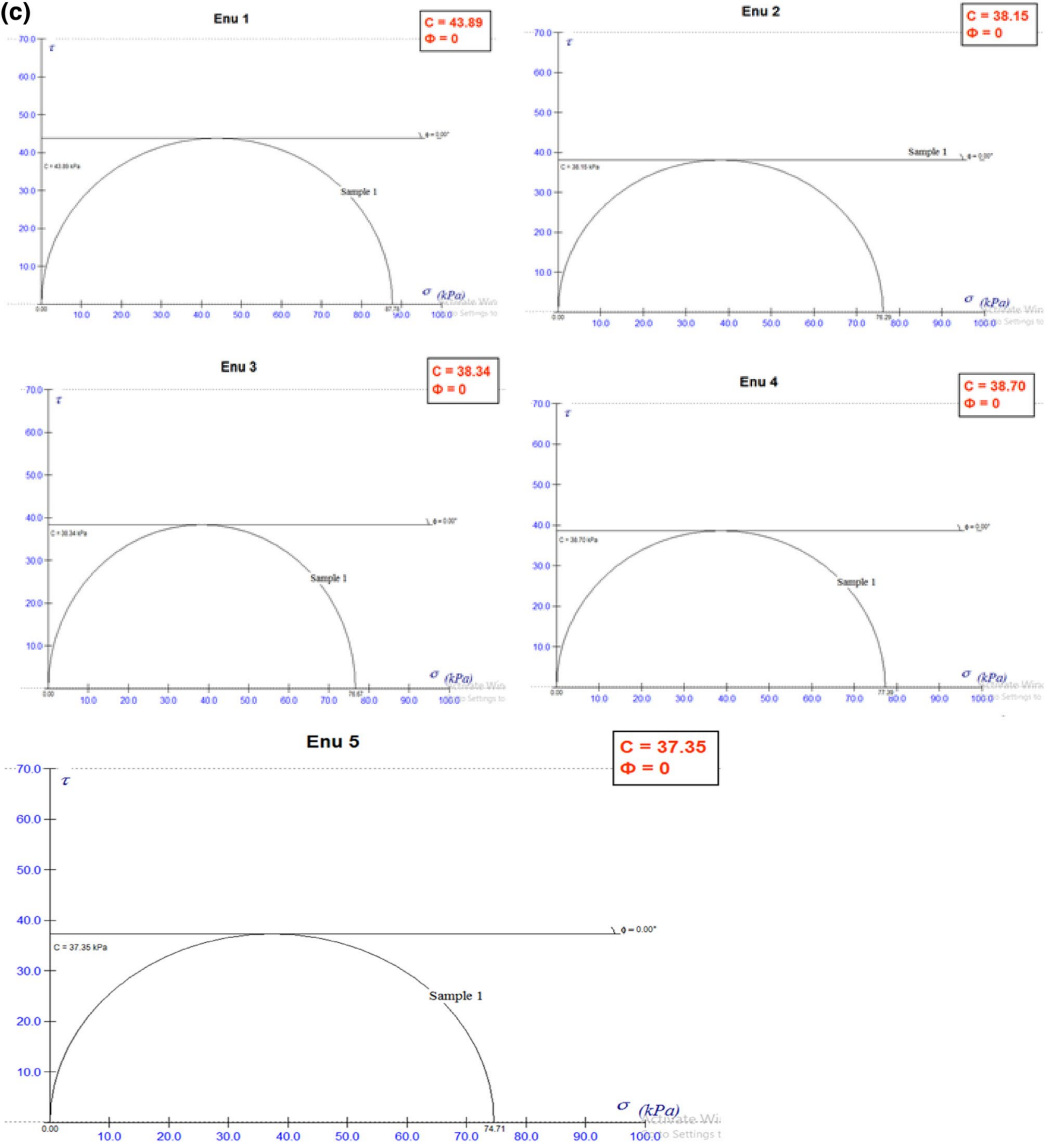

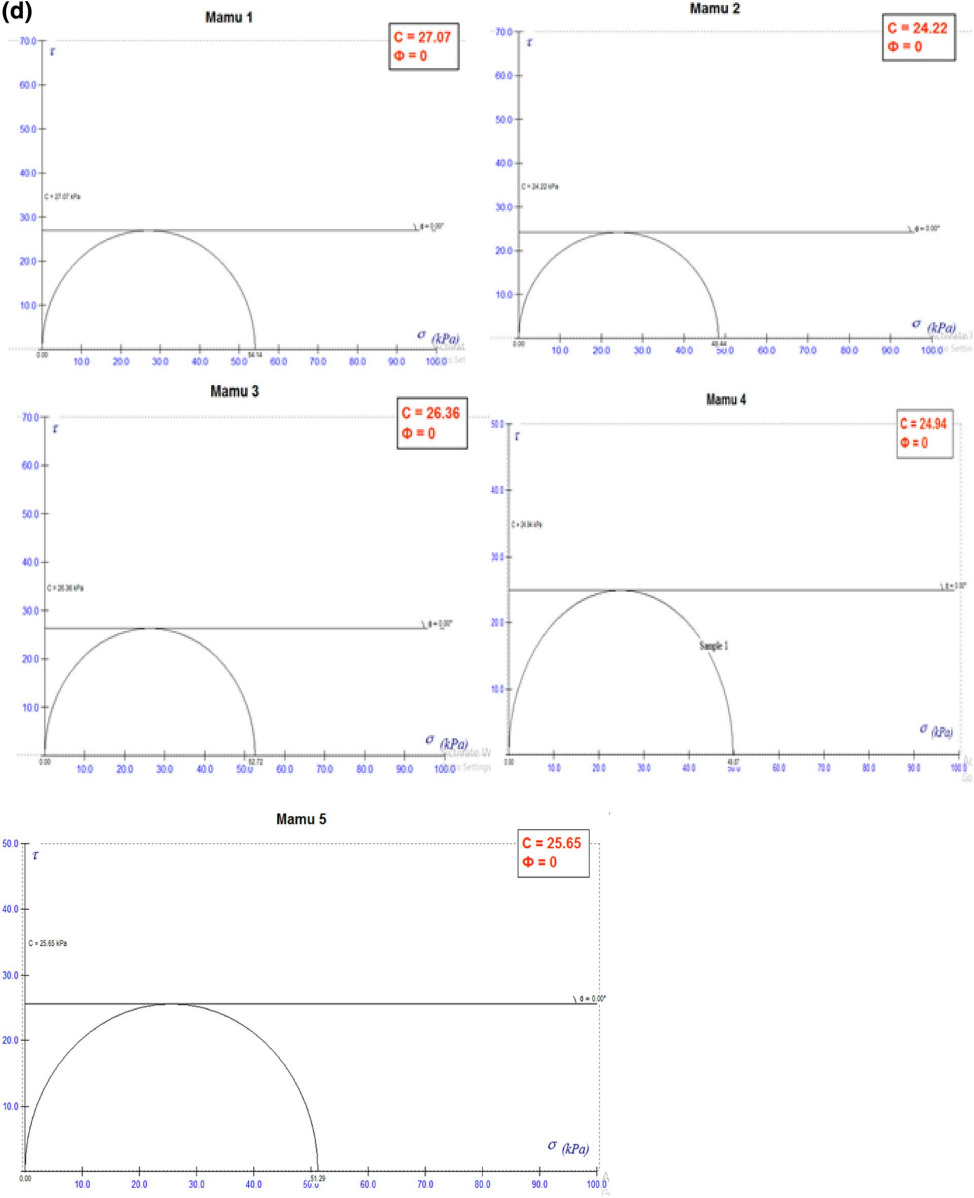
(**a**) Showing the strain curve for Enugu Shale. (**b**) Showing the strain curve for Mamu Shale. (**c**) Failure envelop showing shear strength parameters for Enugu Shale. (**d**) Failure envelop showing shear strength parameters for Mamu Shale.

The analyzed samples revealed values of cohesion (c) ranging from 37.36 to 43.89 kPa for Enugu Shale which was relatively higher than those of Mamu shale with cohesion values ranging from 24.22 to 27.07 kPa, respectively (Table 8; Fig. 5a,b). Both shale samples recorded zero (0) value of the internal frictional angle of shearing resistance (Ф). (Fig. 5c,d). According to Blyth and De Freitas^24^ the reason for high cohesion values may spring from the calcareous nature of the shale. To this end, it is observed that the shear strength of the shale material is determined by the cohesion value. The cohesion values are low, thereby classifying it as a material with low shear strength. Hence, it is not suitable as a good subgrade material except if stabilized and improved.

**Table 8:** Unconfined compressive strength (UCS) result.

Sample ID	UCS (q_u_) at strain 15 (KN/m^2^)	Cohesion (q_u_/2) (c) (KN/m^2^)
E1	87.78	43.89
E2	76.29	38.15
E3	76.67	38.34
E4	77.39	38.70
E5	74.71	37.36
M1	54.14	27.07
M2	48.44	24.22
M3	52.72	26.36
M4	49.87	24.94
M5	51.29	25.65

*E* Enugu Shale,* M* Mamu Shale.

#### California bearing ratio (CBR)

The test outcome revealed that the CBR values of Enugu shales ranged from 1.03 to 1.22%, while those of Mamu shale ranged from 0.90 to 1.60% respectively. These results are way below the standard recommended by the Federal Ministry of Works and Housing Nigeria^21^, which stated that the CBR value for a good subgrade under soaked conditions should be > 5% (Table 5). According to the CBR rating^25^, the materials are rated very poor for subgrade (Tables 9, 10).

**Table 9:** Soil classification based on CBR values (Source Bowels^25^.

CBR (%)	Rating	Objective
0–3	Very poor	Subgrade
3–7	Poor to Fair	Subgrade
7–20	Fair	Subgrade or sub base
20–50	Good	Subgrade or base or sub base
> 50	Excellent	Subgrade or base or sub base

**Table 10:** Soaked CBR test result.

Samples	Soaked CBR (%)	Rating
E1	1.03	Very poor
E2	1.16	Very poor
E3	1.22	Very poor
E4	1.22	Very poor
E5	1.33	Very poor
M1	0.90	Very poor
M2	1.00	Very poor
M3	1.06	Very poor
M4	1.32	Very poor
M5	1.60	Very poor

*E* Enugu Shale,* M* Mamu Shale*.*

### Statistical analysis

#### Correlation analysis

The correlation analysis interpretation utilized the rule of thumb for interpreting the strength of the relationship between two variables based on the regression (r) value (Table 11).

**Table 11:** Absolute value of R^2^ strength of relationship.

R^2^ < 0.25	No relationship
0.25 < R^2^ < 0.5	Weak relationship
0.5 < R^2^ < 0.75	Moderate relationship
R^2^ > 0.75	Strong relationship

##### SDI against PI

**Figure 6:**
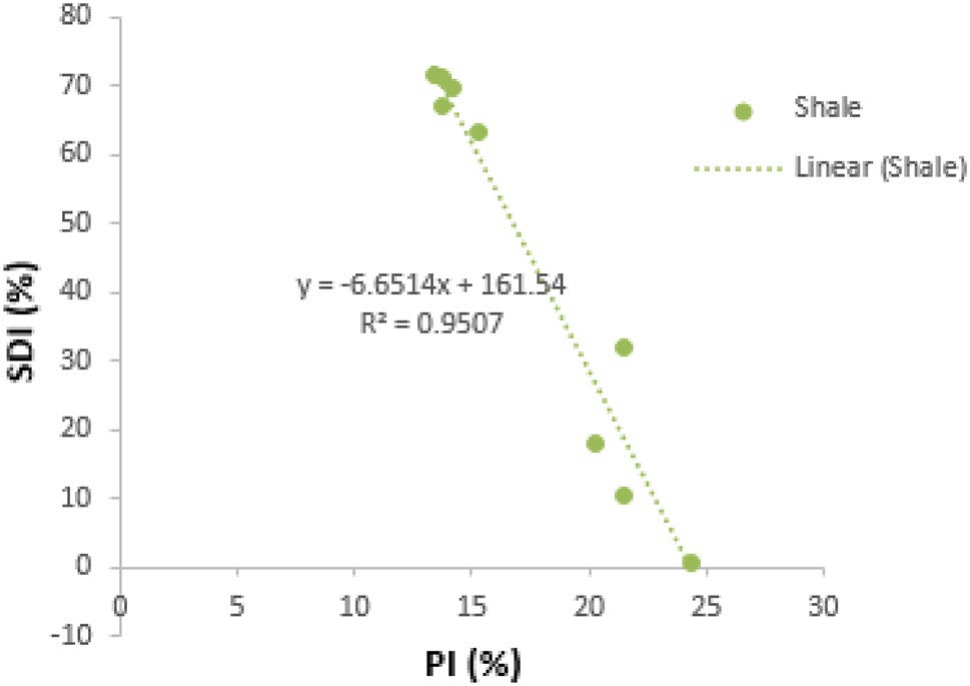
Correlation of SDI against PI for the shale samples.

The regression plot of slake durability index (SDI) against the plasticity index (PI) of the shale samples is presented below for the shale samples. SDI shows a strong inverse correlation against PI, with r value of 0.9507 (Fig. 6). This indicates that as the SDI value increases, the PI value decreases.

##### SDI against CBR

**Figure 7:**
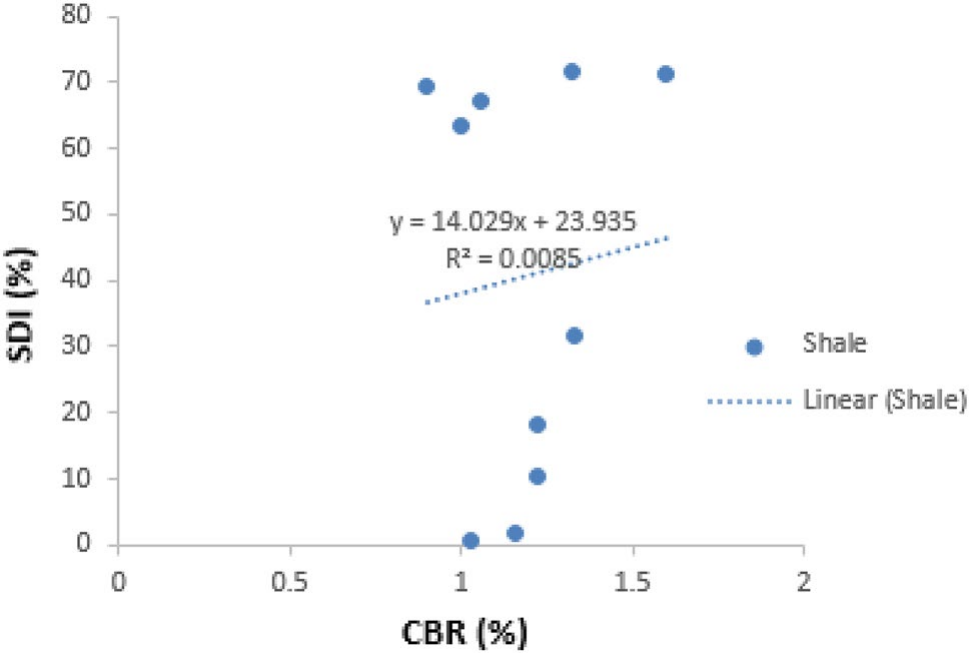
Correlation of SDI against CBR for both Enugu and Mamu shale.

The regression plot of the slake durability index (SDI) against the California bearing ratio (CBR) of the shale samples is presented below (Fig. 7). SDI shows a weak positive correlation against CBR, with r value of 0.0085 for the shale samples. This indicates that the higher the SDI value, the higher the CBR.

##### PI against C

**Figure 8:**
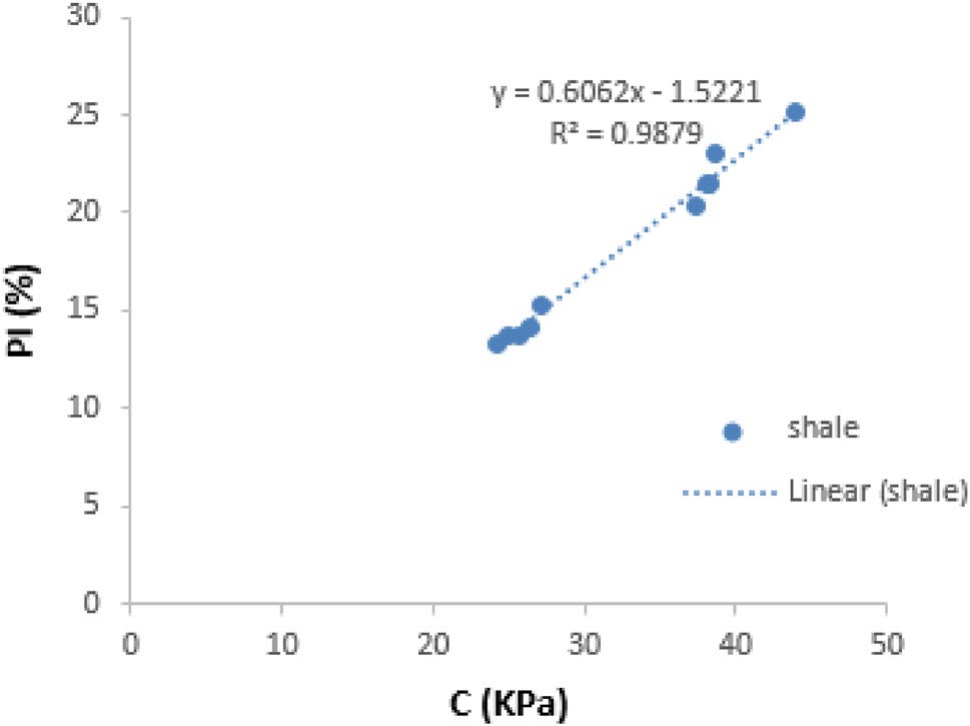
Correlation of PI against C for both Enugu and Mamu shale.

The regression plot of the plasticity index (PI) against the Cohesion (C) of the shale samples is presented below (Fig. 8). PI shows a strong positive correlation against C, with r value of 0.9879 for the shale samples. This implies that the higher the PI values, the higher the C value.

#### Bivariate statistical analysis

The bivariate statistical analysis correlates more than one parameter for easier interpretation. The Pearson correlation of key geotechnical parameters for the shale samples is presented in (Table 12). The correlation is significant at 0.5 levels and not significant if it is less than 0.5 levels. The red-highlighted values indicate that the parameters are positively correlated while the blue-highlighted value indicates that the parameters are inversely correlated. The Pearson correlation of key geotechnical parameters such as SDI, CBR, PI, and C has revealed the relationship between the properties evaluated for shale samples. The Pearson correlation analysis result conforms to the graphical correlation analysis result as presented above.

**Table 12:**
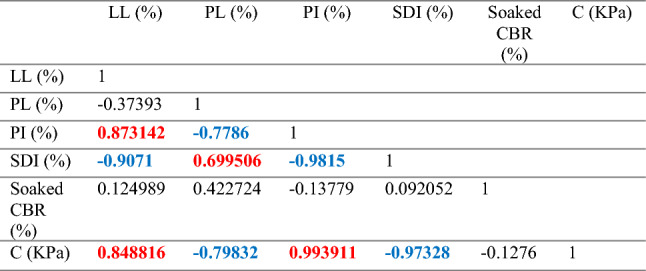
Pearson’s correlation for the shale samples.

Correlation is significant at the 0.5 level.

Where LL (liquid limit), PI (Plasticity Index), PL (plastic limit), SDI (Slake Durability Index), CBR (California bearing ratio) and C (cohesion).

#### X-ray diffraction analysis (XRD) result

**Figure 9:**
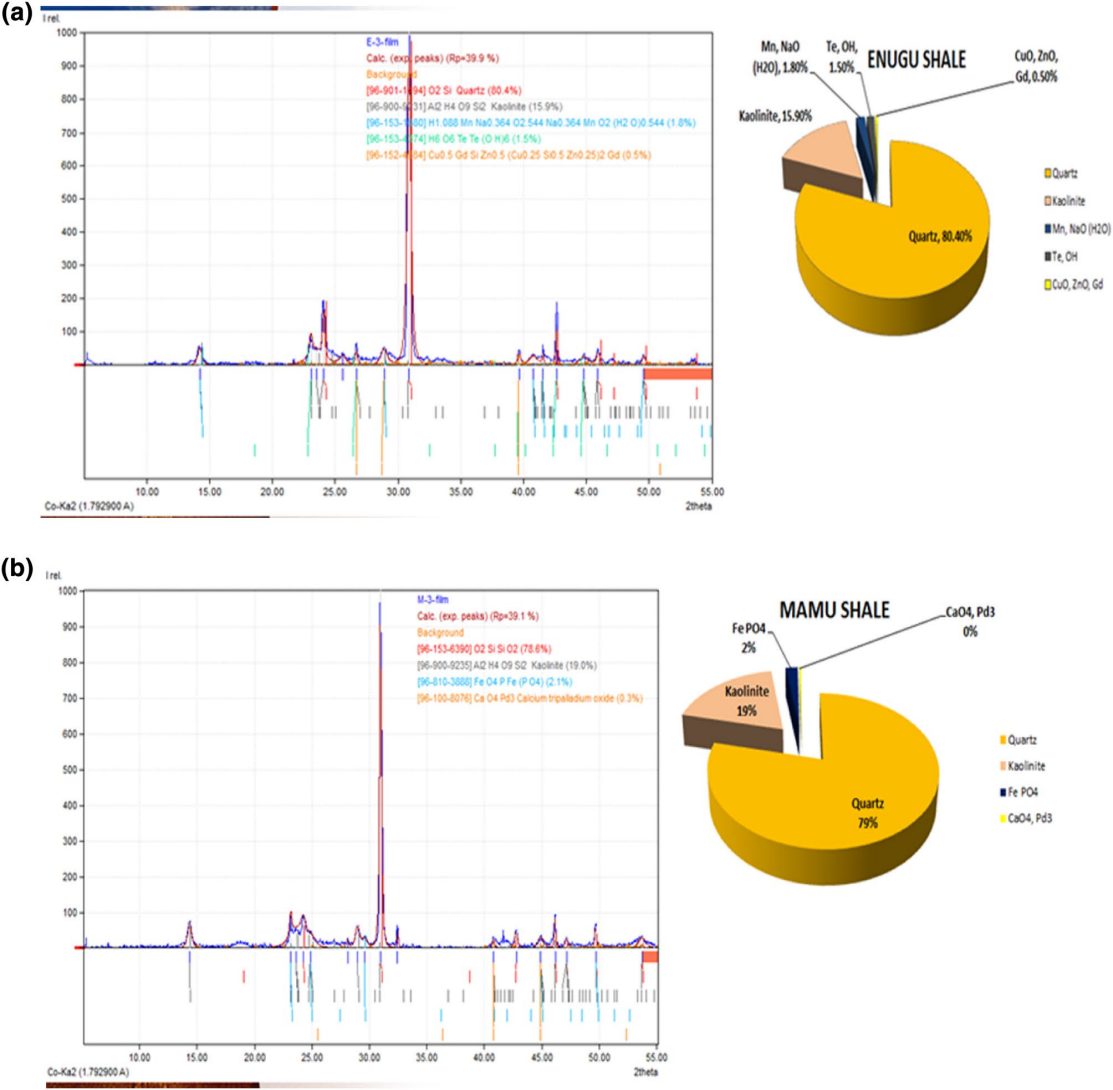
(**a**) XRD section of Enugu shale sample. (**b**) XRD section of Mamu shale sample.

The X-ray diffraction results reveal that the Enugu shale sample is composed mainly of 80.4% Quartz, and 15.9% Kaolinite (clay mineral) with other minor elements such as NaO, MnO_2_, Te, CuO, Gd, and ZnO, making up about 3.7%, while those of Mamu shale is composed mainly of 78.6% Silicon oxide (SiO_2_) and 19.0% Kaolinite (clay mineral) with other minor elements such as Fe, PO_4_, CaO_4_, and Pd_3_, making up about 2.4% (Fig. 9a,b). As displayed, the major clay mineral present in both Shale types is kaolinite. Although kaolinite displays no swelling potential, it can easily be dispersed and migrate in moist conditions^26^, hence leading to pavement failure. This conforms to what was observed in the study area. Furthermore, according to Dumbleton and West^27^, the determination of the plasticity of soil will be of utmost interest in the better understanding of the clay minerals’ performance on engineering structures.

#### Scanning electron microscopy (SEM)

**Figure 10:**
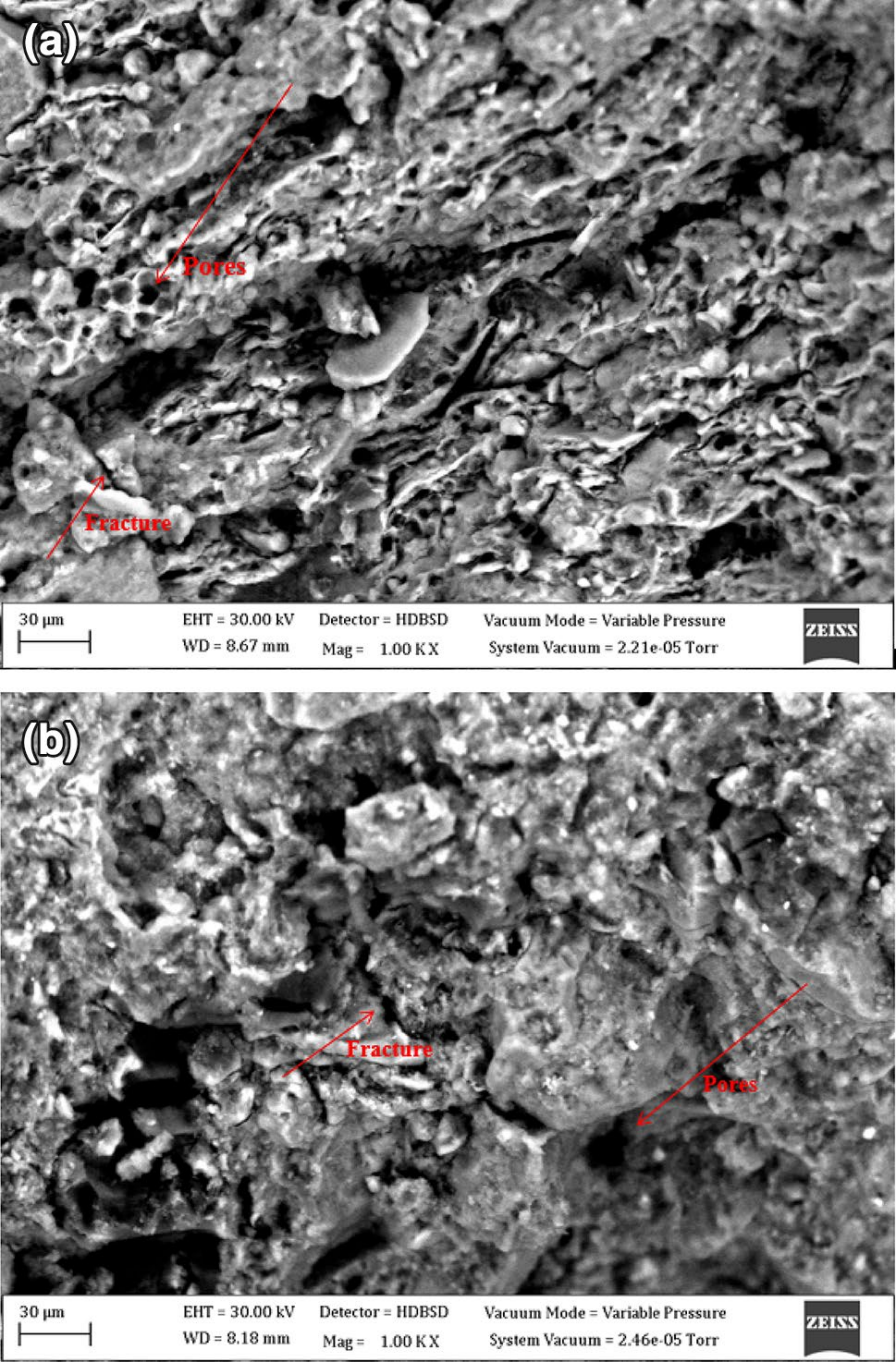
(**a**) Scanning electron microscopic image of Enugu Shale showing micro fractures and pores. (**b**) Scanning electron microscopic image of Mamu Shale showing micro fractures and pores.

The micropores and fractures as revealed by the scanning electron microscope in Fig. 10a,b are enough to induce high moisture content when wet, resulting in mechanical failure by successive wetting and drying processes^28^. The micropores and fractures present give room for water to get accumulated since the material possesses low permeability,this in turn allows pore water buildup and automatically leads to shear strength reduction of the material. Furthermore, the presence of micropores and fractures seen can be used as a reference to why the samples have a relatively high liquid limit, plastic limit with very low to medium slaking, and very low bearing capacity as indicated in the geotechnical test results as reported above.

In addition, the activity of the events that occur within the samples is exposed on the pavement surface as cracks and portholes since it is a flexible pavement. Hence, the shale material is not suitable as a subgrade except if stabilized and treated.

This observation conforms to all geotechnical test carried out for the shale materials gotten from Enugu and Mamu formation.

## Conclusion

Field observation, and geotechnical analysis alongside the mineralogy of both Enugu and Mamu Shales samples has revealed that the shale samples demonstrated characteristics behavior of clay-dominated material (high values of the liquid limit with medium to low plasticity index), which is indeed a contributory factor to the predominant failure of the pavement. The result from the scanned portion of the subgrade displays a large presence of micropores and fractures surrounding the subgrade which makes it vulnerable to physical change via seasonal variation. Although, the samples possess Kaolinite as its major clay mineral with no swelling potential. The kaolinite tends to disperse and migrate in a moist condition which can result in pavement failure.

The test result also reveals that the key geotechnical parameters (LL, PI, SDI, and CBR) that are important for pavement construction for the natural subgrade are way below the recommended standard for the subgrade laid down by the Federal Ministry of Works and Housing Nigeria (FMWH). It is certain that the subgrade geotechnical characteristics are indeed a contributory factor to the constantly damaged road as observed in the study area and therefore do not make good subgrade material.

## Data Availability

The datasets generated during the current study are not publicly available due to (confidentiality agreement between authors to avoid someone else stealing the data to publish another manuscript from it), but data will be readily available from the corresponding author on reasonable request.
